# COMPREHENSIVE REHABILITATION OF A SERVICEMAN WITH TRIPLE LIMB AMPUTATION FOLLOWING COMBAT POLYTRAUMA: A CASE STUDY FROM UKRAINE’S WARTIME CONTEXT

**DOI:** 10.2340/jrm.v57.42950

**Published:** 2025-03-26

**Authors:** Serhii KOLISNYK, FEBPRM, Oleh MURYN, Olha SVYST

**Affiliations:** 1 Department of Physical and Rehabilitation Medicine, National Pirogov Memorial Medical University, Vinnytsia; 2 PRM Physician of Center of Medical Rehabilitation and Sports Medicine, Vinnytsia; 3 Departament of Rehabilitation of Superhumans Center, Lviv; 4 Department of Rehabilitation of Military Medical Clinical Center of the Central Region, Vinnytsia; 5 Head of the Rehabilitation Department of the Central Hospital of the Military Medical Directorate of the Security Service of Ukraine, Kyiv, Ukraine

**Keywords:** multiple amputations, war-related injuries, patient care team, artificial limbs, biopsychosocial model, physical and rehabilitation medicine

## Abstract

The ongoing war in Ukraine has led to a significant rise in combat-related polytrauma injuries, including high-level multiple amputations, delayed evacuations, infectious complications, and limited access to rehabilitation services. This clinical case demonstrates a comprehensive, patient-centred approach to the rehabilitation of a 25-year-old serviceman with triple amputations caused by a mine-blast injury. The patient progressed through pre-prosthetic and prosthetic rehabilitation stages, achieved independent mobility with prosthetics, transitioned to the K3 functional level, and adapted to his independent life. The success of this case underscores the importance of a multidisciplinary approach, modern technologies, and adherence to evidence-based rehabilitation practices. The leading role of the physical and rehabilitation medicine physician was pivotal in coordinating and adjusting the rehabilitation process, ensuring the optimal use of available resources, and aligning the patient’s care with clinical goals. Challenges such as delays in rehabilitation initiation and limited resources highlight the need to expand inpatient rehabilitation capacity and standardize protocols. A coordinated bio-psycho-social rehabilitation model must be the framework for increasing the effectiveness of rehabilitation services and optimizing functioning and quality of life after severe injuries. Implementing multidisciplinary approaches tailored to wartime settings is crucial for combatants’ effective recovery and reintegration.

## LAY ABSTRACT

The war in Ukraine has caused a dramatic increase in severe combat-related injuries, including polytrauma high-level amputations. This case highlights the rehabilitation journey of a 25-year-old serviceman who sustained triple amputations from a blast injury. Through a multidisciplinary, patient-centred approach, the serviceman underwent pre-prosthetic and prosthetic rehabilitation, achieving independent mobility with prostheses and reaching a high functional level of independence. Despite all expectations he regained everyday living activities and new remunerable employment. The role of the physical and rehabilitation medicine physician was crucial in coordinating the process, optimizing resources, extending of indications for prosthetic rehabilitation, and ensuring evidence-based practices. However, challenges like delayed rehabilitation and limited resources emphasize the need for expanded inpatient rehabilitation services and standardized protocols. A bio-psycho-social rehabilitation framework is vital for improving outcomes, especially in wartime conditions. Tailored multidisciplinary approaches are essential for effective recovery and reintegration of combatants and injured civilians.

The Russian–Ukrainian war has led to a surge in complex polytrauma cases, due to modern weaponry, complicated medical care, and battlefield evacuation challenges. While exact statistics on amputations remain classified, the prevalence significantly exceeds international averages, with multiple amputations becoming common, amplifying risks for high-level amputations due to prolonged evacuations, infection risks from blast injuries, and improper tourniquet use, causing complications such as tourniquet syndrome (1).

The rehabilitation system in Ukraine, being relatively new, faces challenges, e.g., a deficit of specialists and an insufficient level of previous appropriate training, that cause inconsistent care quality. Despite prosthetics production and funding advancements, appropriate prescription of relevant functional devices remains uncontrolled. The lack of standardized protocols and coordination during wounded combatant transfers from military to rehabilitation facilities frequently hinders effectiveness and outcomes. This should underscore the need for systemic improvements in indications of prosthetic prescriptions, especially in motivated persons with multiple amputations, and comprehensive specialist training, especially the leading role of physiatrists in rehabilitation teams (2).

This clinical case highlights the success of interdepartmental and multidisciplinary collaboration due to the leading role of a physical and rehabilitation medicine (PRM) physician in achieving comprehensive prosthetic rehabilitation and reintegration of a severely injured individual into independent living and gainful employment during wartime.

## CASE REPORT

**Fig. 1:**
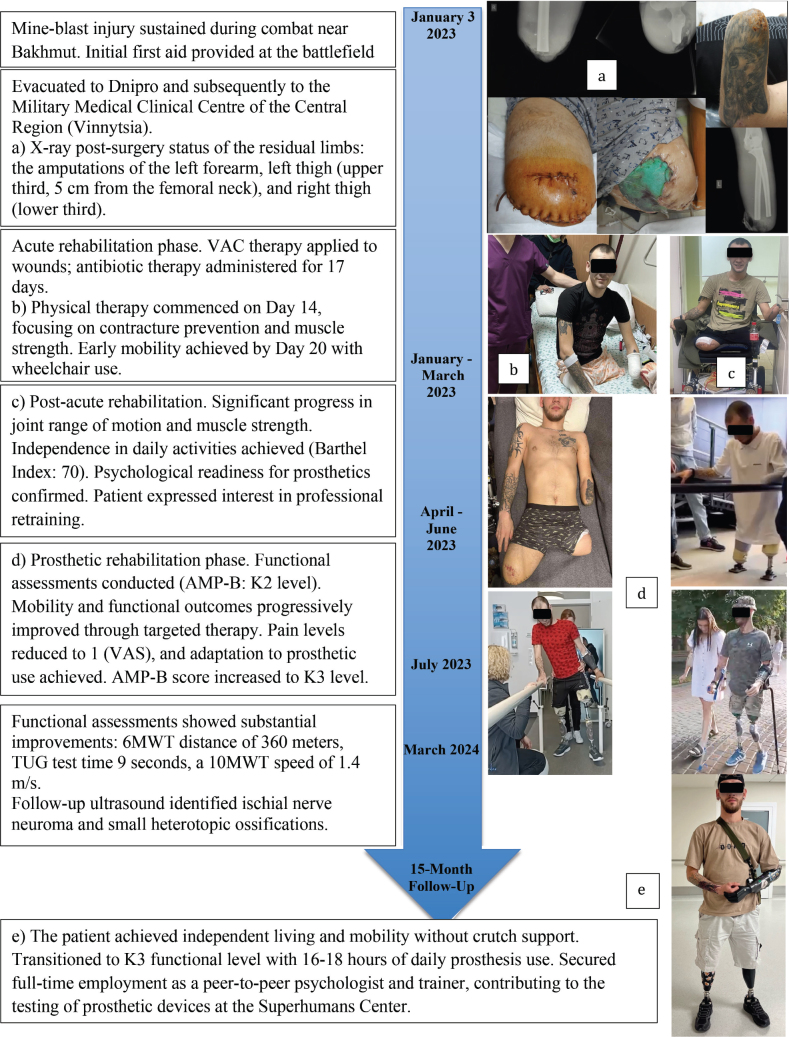
Timeline of the phases of medical and rehabilitative multidisciplinary care of a 25-year-old serviceman with polytrauma and triple limb loss.

A male soldier, born in 1998, sustained a mine-blast injury during combat near Bakhmut on 3 January 2023. Initial first aid was provided on the battlefield and in field hospitals. The patient was subsequently evacuated to Dnipro and then to the Military Medical Clinical Centre of the Central Region (Vinnytsia). Radiological imaging revealed multiple traumatic amputations: the middle third of the left forearm, the upper third of the left thigh (5 cm from the femoral neck), and the lower third of the right thigh (Fig. 1A). The initial clinical evaluation noted severe pain in the stumps (VAS 8/10), phantom pain at 7/10, minor depressive symptoms (PHQ-9 score of 4), mild anxiety (GAD-7 score of 3), and no indications of overexcitement on PTSD screening.

### January–March 2023

Vacuum-assisted closure (VAC) was performed, with antibiotic therapy for 17 days, and there were no complications. Physical therapy comprised contracture prevention, muscle strength and endurance training, PNF, and DNS therapy (Fig. 1B). Occupational therapy included ADL training, scar desensitization and mobilization, compression therapy, and mirror therapy. On day 20 the patient transitioned to using a wheelchair (Fig. 1C).

### April–June 2023

Joint range of motion was normal, and the manual muscle testing (MMT) score was 5/5. The Barthel Index score increased from 30 to 70. Psychological status revealed no signs of anxiety or depression (PHQ-9 and GAD-7), while the Mississippi Combat Stress Assessment Scale yielded a score of 68, indicating a reasonable level of adaptation, and the Mini-Mult assessment revealed no personality disorders. The patient displayed readiness for prosthetic use and expressed interest in professional retraining.

### July 2023–March 2024 (Fig. 1D)

The following resulted were noted: AMP-B tool – functional level K2 (34/47), phantom pain– minimal (VAS: 2/10); mobility testing: Timed Up and Go (TUG) test – 19 s (using 2 crutches and prostheses), a 6-Minute Walk Test (6MWT) – 235 m with heart rate of 145 bpm, a Four Square Test (FST) time of 17 s, 10-Meter Walk Test (10MWT) at 1 m/s, and MMT for all muscles was 5/5. An individualized rehabilitation plan was created based on the ICF approach, acknowledging the absence of specific ICF Core Sets for patients with multiple amputations.

### September 2023

Moderate progress was seen: the AMP-B score increased to 36, maintaining the K2 level, TUG – 16 s using a single elbow crutch, 6MWT – 255 m, with a heart rate of 148 bpm post-test, the FST – 12 s. The Stair Climb Test produced a descent time of 36 s, and an ascent time of 29 s while using handrails, and the 10MWT was completed at 1.1 m/s.

### January–March 2024

VAS score was 1/10. The AMP-B score improved from 36 (K2) to 39 (K3), 6MWT showed an increase to 360 m while using a left-hand prosthesis and crutches. The Stair Climb Test demonstrated significant progress, with a descent time of 9 s and an ascent time of 15 s. 10MWT speed improved to 1.4 m/s. The Locomotor Capabilities Index-5 score reached 45/56, and the Houghton Scale score was 9/12. Additional findings from the ultrasound in March 2024 included the presence of an ischial nerve neuroma (9 mm, minimally painful), small heterotopic ossifications (3–5 mm), and multiple soft tissue shrapnel fragments (4.4 mm).

### Fifteen months outcomes (Fig. 1E)

The patient achieved independent living, mobility, and ADLs using prosthetics without crutch support. He transitioned to K3 functional level, enabling a higher activity level and an improved quality of life with 16–18 h of prosthesis use daily. Psychological adaptation was successful, and he pursued professional retraining, becoming a peer-to-peer psychologist. The patient secured full-time remunerative employment as a first-contact trainer-instructor and peer-to-peer counsellor and became an expert in testing new prosthetic devices at the Superhumans Center.

## DISCUSSION

Despite the multiple experiences of acute wartime amputations, the outcomes were unexpected. The rehabilitation approach was built on main evidence-based principles and the bio-psycho-social model. A patient-centred approach was implemented, considering environmental and personal factors, and all treatment and rehabilitation stages were planned according to the patient‘s needs, capacities, and wishes, with active patient involvement in decision-making (3, 4). The multidisciplinary approach, coordinated teamwork, and a staged and continuous SMART model for goal-setting and planning ensured optimal functional outcomes. Early rehabilitation activities and gradual progression to more complex tasks promoted a swift recovery.

Pre-prosthetic treatment was conducted at a high level without any infectious complications. VAC therapy, appropriate antibiotics, and early mobilization provided optimal conditions for primary prosthetics. The use of modern technologies, such as a bionic prosthesis, in combination with the patient’s preparation, allowed for a faster return to an active life. Psychosocial team support, managed by a physiatrist, including psychologists’ interventions, preventing the re-traumatization, and the patient’s early professional retraining, was a significant motivating factor in social reintegration. However, earlier initiation of acute rehabilitation in an intensive care unit in a military hospital could have facilitated a faster transition to the post-acute rehabilitation stage. This emphasizes the importance of access to specialized rehabilitation resources and integrating rehabilitation measures into the overall treatment process, particularly in security and defence forces facilities.

In this context, the role of PRM physicians was critical (2). The PRM physician managed and coordinated rehabilitation efforts and played a pivotal role in adapting the rehabilitation process to the available resources and infrastructure, especially under wartime conditions.

This clinical case illustrates the need to expand rehabilitation capacity, providing early holistic rehabilitation in Ukrainian military hospitals. It also highlights the leading role of PRM physicians in optimizing combatant outcomes and extending the indications for prosthetic rehabilitation in multiple amputations in young persons who would not have been supported without appropriate funding.

## CONCLUSION

1. The clinical cases of patients with blast polytrauma involving multiple acute limb losses are characterized by extensive tissue damage, complex wounds with shrapnel presence, concomitant systemic injuries, and neurological and internal organ trauma, necessitating comprehensive investigations to develop consensus for enhancement of rehabilitation strategies and patient outcomes.
2. Although the severity of injuries poses significant challenges, employing multidisciplinary, interdisciplinary, and transdisciplinary rehabilitation approaches can optimally facilitate recovery, restore functional independence, and promote reintegration into active life and employment, particularly in countries with evolving rehabilitation systems under wartime conditions.
3. To address the extensive variability in rehabilitation quality across different facilities, robust management protocols that incorporate a holistic team approach led by PRM physicians in the development of individualized rehabilitation plans are imperative.
4. In the wartime condition rehabilitation system in Ukraine, which is rapidly developing, adopting a multidisciplinary approach shows promise but necessitates additional research and tailored strategies to confront the unique challenges.
5. The expansion of prosthetic rehabilitation criteria to encompass multiple traumatic amputations warrants further investigative studies to optimize treatment pathways and improve patient outcomes.
